# Normothermic Kidney Perfusion: Current Status and Future Perspectives

**DOI:** 10.1016/j.ekir.2025.06.041

**Published:** 2025-06-26

**Authors:** Barbara Franchin, Leonie van Leeuwen, Matthew L. Holzner, Nicholas Chun, Lucrezia Furian, Paolo Cravedi

**Affiliations:** 1 Renal Division, Department of Medicine, Translational Transplant Research Center, Icahn School of Medicine at Mount Sinai, New York, New York, USA; 2 Recanati/Miller Transplantation Institute, Icahn School of Medicine at Mount Sinai, New York, New York, USA; 3 Kidney and Pancreas Transplantation Unit, Department of Surgery, Oncology and Gastroenterology DISCOG, University Hospital of Padova, Padova, Italy

**Keywords:** *ex situ* perfusion, graft recovery, ischemia-reperfusion injury, kidney transplantation, marginal donors, target-therapies

## Abstract

Normothermic machine perfusion (NMP), the most recent advancement in solid organ preservation, enables *ex situ* maintenance of grafts in a physiologically active state, offering a significant advantage over traditional cold storage methods. Whereas NMP is now widely adopted for clinical preservation of livers, hearts, and lungs, its application in kidney transplantation remains relatively limited. In this context, NMP holds promise for expanding the use of marginal donor kidneys by enhancing viability assessment and potentially restoring function in grafts that might otherwise be discarded. In addition, NMP provides a valuable platform for studying molecular markers of injury and recovery in human organs, as well as for delivering targeted therapies aimed at modulating immunologic or transcriptomic profiles. Despite its potential, broader clinical implementation is hindered by variability in perfusion devices, protocols, and perfusate compositions across centers, making cross-study comparisons challenging. This review examines the current landscape of kidney NMP and its emerging role in graft reconditioning.

For decades, static cold storage (SCS) has remained the gold standard for organ preservation because of its simplicity and cost-effectiveness.^1^ However, the ongoing organ shortage and increasing reliance on marginal donors have spurred the development of alternative strategies to improve graft viability. Notably, the use of kidneys from donors after circulatory death (DCD) continues to increase, reflecting the growing prevalence of higher-risk grafts. In 2024, DCDs reached 20.6% of deceased donors in Eurotransplant and 43% in the United States^2^^,^^3^; whereas in 2020, they represented 17.1% and 25.6% of all deceased donor, respectively.^4^^,^^5^ Compared with donors after brain death, DCD kidneys experience prolonged donor warm-ischemia-time, which is associated with higher rates of delayed graft function (DGF), greater susceptibility to ischemia-reperfusion injury, and increased risk of early graft loss.^6^ Furthermore, donors of advanced age and with comorbidities, especially hypertension and diabetes, increase the risk of DGF and early graft loss in both donation after brain death and DCD kidneys.^7^ This has driven interest in advanced preservation techniques to try and mitigate the vulnerabilities of these organs.

Hypothermic machine perfusion (HMP), which preserves the organs at 4 °C, while maintaining continuous perfusion, has emerged as a valuable alternative to SCS.^8^ A landmark multicenter European trial demonstrated that nonoxygenated HMP significantly reduced DGF (adjusted odds ratio: 0.57; *P* = 0.01) and graft loss compared with SCS and was associated with improved 1-year graft function.^9^ Nowadays, HMP is widely used for preserving deceased donor kidneys in many countries, including the United States.

NMP offers a more physiological approach by maintaining organs at body temperature in an oxygen- and nutrient-rich environment, preserving metabolic activity, and allowing real-time functional assessment. The first clinical kidney transplant using NMP was performed in 2011 as a head-to-head trial in which the 2 kidneys from 1 donor were treated with SCS alone or SCS and 35 minutes of NMP. Both organs were successfully transplanted, and the NMP-treated kidney showed no DGF and superior function at 3 months posttransplant.^10^

Whereas NMP is now routinely used for liver, heart, and lung preservation, its application to kidneys remains largely experimental because of technical complexities specific to renal perfusion.^11^ Still, kidney NMP holds great promise for enhancing graft assessment, enabling targeted interventions, and improving both allocation strategies and post-transplant outcomes.^12^^,^^13^

This review explores the development of the clinical application of kidney NMP, its role in graft function assessment, and its potential to increase organ utilization rates and modulate graft immunogenicity.

A comprehensive literature search was conducted on PubMed without time restrictions, with the final search completed in February 2025. The following searching terms were used with “AND” and “OR”: normothermic machine perfusion, kidney, transplant, kidney transplantation, and normothermic perfusion.

### Preclinical and Clinical Evidence for Kidney NMP

NMP has been extensively explored for its safety, feasibility, and potential to assess viability and recondition marginal kidneys in a preclinical setting (Table 1).^14^, ^15^, ^16^, ^17^, ^18^, ^19^ In a porcine DCD autotransplant model, Kaths *et al.*^14^ showed that 8-hour NMP was safe and significantly improved graft function compared with SCS alone. These benefits were further validated in models with prolonged warm-ischemia-time, where NMP was associated with lower peak serum creatinine and faster recovery.^18^ Additional studies in porcine models explored the optimal duration of NMP. Although the potential benefits of extended perfusion times for graft reconditioning remain under debate, they pose increased technical challenges and raise the risk of metabolic waste accumulation if appropriate long-term NMP protocols are not carefully implemented.^15^^,^^20^ In a direct comparison of 1, 8, and 16 hours of NMP, Kaths *et al.*^16^ found that intermediate and prolonged NMP resulted in superior posttransplant function versus brief NMP, which was actually inferior to SCS alone. Similarly, Urbanellis *et al.*^19^ demonstrated that continuous 16-hour NMP outperformed both SCS and HMP in early renal function.

**Table 1:** Preclinical NMP studies

Author	Center	Species	Case number	Device	Protocols	Time on NMP (h)	T (°C)	MAP initial (mmHg)	MAP maintenance (mm Hg)	Organ viability assessment	Dosed biomarkers on perfusate
Kaths et al.^14^	Toronto General Hospital, Ontario, Canada	Pig	10	Not commercial	NEVKP	8	37	75	65	Acid-base homeostasis	LDH, AST
Kaths et al.^15^	Toronto General Hospital, Ontario, Canada	Pig	15	Not commercial	SCS, NEVKP	- 16 SCS - 15 SCS + 1 NEVKP - 8 SCS + 8 NEVKP - 16 NEVKP	37	75	65	Perfusion flow, acid-base homeostasis, electrolyte concentrations	LDH, AST
Kaths *et al.*^16^	Toronto General Hospital, Ontario, Canada	Pig	15	Not commercial	SCS, NEVKP	- 8 SCS - 8 SCS + 1 NEVKP - 8 SCS + 8 NEVKP - 8 SCS + 16 NEVKP	37	75	65	Acid-base homeostasis	LDH
Weissenbacher *et al.*^17^	Institute of Biomedical Engineering, University of Oxford, Oxford, UK	Human	11	Not commercial	NMP with vs. without urine recirculation	24	37	70–100	70–100	Acid-base homeostasis , histology	NGAL, KIM-1
Urbanellis et al.^18^	Toronto General Hospital, Ontario, Canada	Pig	11	Not commercial	NEVKP	8	37	60–80	60–80	Perfusion flow, acid-base homeostasis, electrolyte concentrations	-
Urbanellis *et al.*^19^	Toronto General Hospital, Ontario, Canada	Pig	15	Not commercial	NEVKP	16	37	75	65	Perfusion flow, acid-base homeostasis, electrolyte concentrations	LDH, AST

AST, aspartate transferase; KIM-1, kidney injury molecule-1; LDH, lactate dehydrogenase; MAP, mean arterial pressure; NEVKP, normothermic ex vivo kidney perfusion; NGAL, neutrophil gelatinase-associated lipocalin; NMP, normothermic machine perfusion; SCS, static cold solution; T, temperature.

Human studies thus far have primarily focused on safety and feasibility; nevertheless, the results support the potential benefits of NMP (Table 2).^10^^,^^21^, ^22^, ^23^, ^24^, ^25^, ^26^, ^27^, ^28^ Most commonly, kidneys are transported using established preservation techniques (HMP or SCS) and undergo NMP upon arrival at the transplant center. This staged approach has proven feasible and safe. In a North American cohort, combining HMP with short-term NMP (median 171 minutes) showed a trend toward reduced DGF compared with HMP alone.^23^ In the Eurotransplant region, Rijkse *et al.*^22^ reported a significant reduction in DGF after 2 hours of NMP compared with historical HMP-only controls; whereas in Australia, a paired study of 18 kidneys showed that 1 to 3 hours of NMP reduced DGF rates (23.5% vs. 64.7%) compared with SCS, although there were no differences in graft function or survival at 1 year.^25^

**Table 2:** Clinical NMP studies

Author	Center	Species	Case number	Device	Protocol	Time on NMP (min)	T (°C)	MAP (mm Hg)	Organ viability assessment	Dosed biomarkers (perfusate)	Transplant performed	DGF NMP group	DGF control group
Hosgood and Nicholson^10^	Leicester General Hospital, Leicester, UK	Human	2	Not commercial	SCS, NMP	35	33.9 ± 0.6	65–70	Macroscopic appearance	-	Yes	0/1	1/1
Minor *et al.*^21^	University Hospital Essen, Essen, Germany	Human	1	Kidney Assist (Organ Assist)	COR	120	35	75	Perfusion flow, electrolyte concentrations	-	Yes	0	-
Rijkse *et al.*^22^	ErasmusMC University Medical Centre, Rotterdam, the Netherlands	Human	11	Kidney Assist (Organ Assist)	HMP^a^, NMP	120	37	60	Perfusion flow, acid-base homeostasis, electrolyte concentrations	-	Yes	4/11	28/53
Mazilescu *et al.*^23^	Toronto General Hospital, ON Canada	Human	39	Not commercial	HMP, NMP	171 (44–275)	37	75 until rewarming then 65	Perfusion flow, acid-base homeostasis	-	Yes	4/13	10/26
Hosgood *et al.*^24^	Addenbrooke’s Hospital, Cambridge, UK	Human	277	Not commercial	SCS, NMP	60	35–37	70–85	Quality assessment score	-	Yes	82/135	83/142
Hameed *et al.*^25^	Westmead Hospital, Sydney, NSW, Australia	Human	35	Kidney assist organ perfusion system (XVIVO Abdominal)	NMP	60 (5–120)	37	75	Perfusion flow, acid-base homeostasis, electrolyte concentrations	-	Yes	5/18	11/17
Dumbill *et al.*^26^	Oxford University Hospitals NHS Foundation Trust	Human	36	OrganOx Ltd	SCS^a^, NMP	348 (132–1404)	36.7 ± 1.5	87.4 ± 5.2 74.8 ± 0.2	Macroscopic appearance	NGAL, KIM-1, L-FABP, GST, LDH, AST, IL-18, cell-free DNA	Yes	13/36	27/72
NCT 06263023 (OPTIMAL) enrollment completed^27^	Purdue Research Park of West Lafayette	Human	-	-	Sub-NMP	-	25	-	Perfusion flow, oxygenation, electrolyte concentrations, quality assessment score	-	Yes	-	-
NCT 04882254 (APOLLO) ongoing^28^	Erasmus MC University Medical Centre, Rotterdam, the Netherlands	Human	-	-	NMP	120	37	-	-	-	Yes	-	-

AST, aspartate transferase; COR, controlled oxygenated rewarming; DGF, delayed graf function; GST, glutathione S-transferases; HMP, hypothermic machine perfusion; IL, interleukin; KIM-1, kidney injury molecule-1; LDH, lactate dehydrogenase; L-FABP, liver fatty acid-binding protein; MAP, mean arterial pressure; NGAL, neutrophil gelatinase-associated lipocalin; NMP, normothermic machine perfusion; SCS, static cold storage; T, temperature.

a Matched historical cohort.

A recent randomized controlled trial by Hosgood *et al.*^24^ evaluated 290 DCD kidneys preserved with either SCS alone or SCS followed by 1-hour NMP using an oxygenated red blood cell–based solution. Although confirming the safety of NMP, the study showed no significant difference in DGF, rejection, graft function, or survival; findings attributed to the short NMP duration and the proinflammatory effects of red blood cell–based perfusates, which is consistent with the preclinical observations that a threshold duration of perfusion may be required to achieve functional benefits.

Of note, even short NMP (2 hours) affects the graft metabolic pathways; analysis performed on discarded human kidneys showed a shift toward oxidative phosphorylation and adenosine triphosphate production whereas complement and coagulation factor synthesis were downregulated. However, the functional impact of these findings is not known yet.^29^

Weissenbacher *et al.* demonstrated that stable 24-hour perfusion of discarded human kidneys is feasible by recirculating the produced urine back into the perfusate, permitting the first prolonged NMP trials in humans.^17^ Dumbill *et al.*^26^ performed a phase 1 trial using up to 24 hours of oxygenated NMP following SCS. Though the approach was safe and feasible, there were no differences in DGF, graft function, or survival compared with SCS-only controls. Unlike animal models, the extended NMP duration did not yield functional advantages, likely because of differences in donor quality and prolonged initial SCS (mean: 9 hours), which may have blunted the benefits of NMP.

Beyond improving graft quality, prolonged NMP may offer logistical advantages, such as extended preservation time, reduced discard due to prolonged ischemia, and improved surgical scheduling (i.e., reduced overnight transplants). These findings collectively support further clinical trials of NMP to determine optimized timing, perfusate composition, and donor selection criteria to maximize benefit.

### Kidney NMP Setup and Perfusate Composition

Although there have been several clinical studies on kidney NMP, there is no standardization in the field. Current protocols vary widely in terms of perfusion duration, device type, hemodynamic parameters, and perfusate composition, impacting *ex situ* kidney function, and likely posttransplant outcomes.^30^

#### NMP Device and Circuit Design

A standard kidney NMP circuit typically consists of a perfusion pump, either a custom designed pump or a repurposed cardiopulmonary bypass machine (Figure 1). This pump may deliver continuous or pulsatile flow and can be configured for pressure- or flow-controlled perfusion. The circuit can be an open (only artery cannulated) or closed (artery and veins cannulated system), consisting of tubing, an organ chamber (retains moisture), oxygenator, and heat exchangers or heating pads to maintain normothermic temperatures. The system can also incorporate urine recirculation,^17^ which minimizes electrolyte and fluid loss, and has been shown to be crucial for maintaining homeostasis during extended perfusions.^30^ In addition, perfusate and urine collection ports allow for real-time biochemical monitoring (e.g., pH, lactate, and electrolytes), and sensors to help regulate relevant perfusion parameters (e.g., pressure, flow, and temperature). Some devices are portable and can be brought to a procurement center, whereas the majority are designed to be deployed once the organ reaches the transplant center (back to base). All these system-specific factors influence graft function and biomarker profiles; however, understanding exactly how manipulating one parameter affects graft health remains a challenge because of the number of parameters and the inherent variability of the pumped organs.

**Figure 1:**
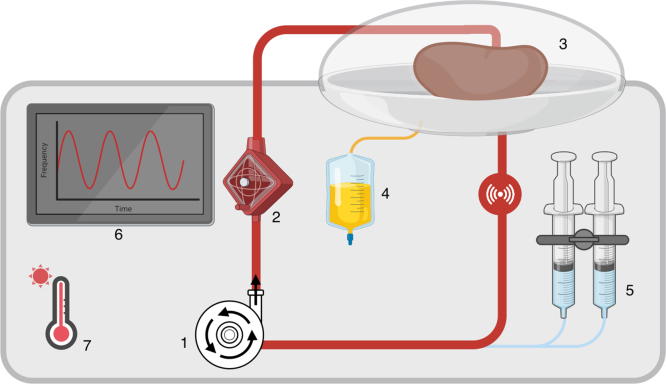
Schematic representation of NMP circuit. (1) Continuous or pulsatile pump, (2) oxygenator, (3) organ chamber, (4) urine collector, (5) infusions, (6) screen providing hemodynamic parameters measured by in line sensors, and (7) Heater. NMP, normothermic kidney perfusion.

#### Perfusate Composition and Other Considerations

Effective NMP of the kidney hinges on the continuous delivery of oxygen, nutrients, and electrolytes to preserve cellular function and organ viability. Although red blood cell–based perfusates remain the most widely used because of their physiological oxygen-carrying capacity and demonstrated efficacy in preclinical models, their reliance on compatible blood products presents logistical challenges.^31^

STEEN solution, originally formulated for *ex situ* lung perfusion, has been adapted for renal NMP and shown to support prolonged perfusion, both with and without red blood cell supplementation.^32^^,^^33^ In a landmark study, Minor *et al.*^21^ reported the first successful kidney transplant following controlled oxygenated rewarming (COR) using STEEN solution saturated with 100% oxygen, suggesting that adequate oxygenation may be achievable without traditional carriers, at least over shorter perfusion durations.

Alternative oxygen delivery strategies, such as synthetic hemoglobin-based oxygen carriers, have also been evaluated in discarded human kidneys.^34^^,^^35^ These studies indicate that hemoglobin-based oxygen carriers can effectively support renal perfusion and oxygenation. However, prolonged NMP using perfusates based on hemoglobin-based oxygen carriers has been associated with methemoglobin accumulation, which may impair oxygen delivery.^36^ Further studies are required to determine the safety and efficacy of hemoglobin-based oxygen carriers across various perfusion durations and settings.

Emerging evidence suggests that differences in perfusate composition can significantly affect renal function, injury marker profiles, and electrolyte balance during NMP; however, systematic comparisons remain limited given the confounding effects of different NMP set-ups and protocols noted above.^30^^,^^37^^,^^38^

Dissecting the interplay between these variables will be essential for optimizing NMP performance and moving toward clinical implementation in kidney transplantation.

### Graft Assessment During NMP

#### Quality Assessment Score

In liver and lung transplantation, NMP is a platform for assessing graft function and enabling organ reconditioning. However, functional assessment of kidney grafts is not standardized and there is a lack of reliable predictive markers.

To address this gap, Hosgood *et al.*^39^ developed an *ex situ* scoring system—the Quality Assessment Score (QAS)—to evaluate kidney graft viability after 1 hour of NMP. Discarded human kidneys were assessed based on the following 3 key parameters:

1. Macroscopic kidney evaluation: excellent perfusion (grade I); moderate perfusion (grade II); poor perfusion (grade III)
2. Renal blood flow (ml/min per 100 g): ≥ 50 vs. < 50
3. Total urine output (ml): ≥ 43 vs. < 43

These criteria generate a composite score from 1 (minimal injury) to 5 (severe injury). Kidneys scoring between 1 and 3 were considered suitable for transplantation, whereas those scoring 5 were deemed high-risk and unsuitable. This scoring system was validated in a clinical cohort of 36 NMP-preserved kidneys, all of which had QAS scores between 1 and 3.^39^ Notably, the overall rate of DGF was lower than expected and mostly associated with score 3 that also correlated with higher posttransplant creatinine levels at 12 months. This highlights NMP’s predictive value of the score and its role in decision making process.

Importantly, the QAS proved useful in assessing potential donor organ resuscitation. Following NMP, 5 of 10 organs that had been previously declined by all UK transplant centers because of poor *in situ* perfusion achieved a QAS of 1 to 3 and were successfully transplanted.^13^ Four exhibited immediate function, whereas 1 developed DGF and showed suboptimal function at 6 months; thereby demonstrating the potential of NMP and QAS to rescue and assess marginal grafts that might otherwise be discarded. To our knowledge, there are currently no other assessment scores used to assess *ex situ* kidney function before transplantation.

#### Biomarkers

Several biomarkers of kidney injury have been explored in both HMP and NMP; however, a reliable and standardized set of injury and recovery markers remains elusive. Although NMP replicates many aspects of *in vivo* physiology, it does not mimic all the body systems (e.g. endocrine, immune, and musculoskeletal), which may in part explain why the biomarkers routinely used to assess renal injury and recovery *in vivo* showed limited utility in the *ex situ* setting.^40^

Neutrophil gelatinase-associated lipocalin and kidney injury molecule-1, 2 of the most established markers in native kidney disease and posttransplant injury, show inconsistent predictive value in the context of NMP.^25^^,^^40^^,^^41^ Neutrophil gelatinase-associated lipocalin correlates with donor serum creatinine, metabolic indicators such as lactate and pH, and other injury markers such as kidney injury molecule-1 and liver fatty acid binding protein.^13^ However, it performs poorly as a standalone predictor of DGF, and may have greater utility when integrated into multimarker signatures.^40^^,^^41^ Kidney injury molecule-1, though recognized as a sensitive biomarker of injury in native kidneys and a predictor of graft loss following transplantation,^42^ has demonstrated paradoxical associations with graft function during NMP, including correlations with improved outcomes in some studies.^25^^,^^43^

More recently, alternative biomarkers have emerged, including glutathione S-transferase pi, an enzyme involved in antioxidant defense. Elevated glutathione S-transferase pi levels in perfusate have been associated with a higher risk of DGF; paradoxically, however, persistent elevation of glutathione S-transferase pi has also been linked to improved long-term graft outcomes.^26^ Endothelin-1, a vasoactive peptide, has demonstrated potential as an ischemic injury marker in both animal and human models, with associations observed between urinary endothelin-1 levels, neutrophil gelatinase-associated lipocalin, and histological injury.^41^^,^^43^Flavin mononucleotide, a mitochondrial stress marker, correlates with posttransplant renal function in early clinical studies; however, it showed no correlation with QAS.^44^ Together, these findings highlight the promise of several novel biomarkers; however, current evidence remains fragmented and at times contradictory. Larger prospective clinical studies are necessary to validate the predictive utility of these biomarkers and to clarify how individual markers or composite panels such as integration with QAS could inform graft evaluation and decision-making during *ex situ* perfusion.

### *Ex situ* Immunomodulation and Targeted Therapies

NMP represents a transformative advancement in organ preservation, offering the ability to assess and recondition grafts prior to transplantation. However, it may also affect immune responses. Unlike SCS, NMP initiates reperfusion injury *ex situ*, creating a proinflammatory environment that may be compounded by exposure to synthetic circuit components; paralleling effects seen in extracorporeal membrane oxygenation and dialysis. These processes can activate the complement cascade and promote oxidative stress, potentially priming the graft for heightened immune recognition posttransplantation.^6^

#### Innate Immune Activation During NMP

Jager *et al.*^45^ demonstrated that NMP triggers rapid complement activation in both porcine and human kidneys, with marked increases in C3d and membrane attack complex following NMP. This response was attributed to both exogenous material contact and reduced complement inhibition in the *ex vivo* environment. In addition, the study found a direct correlation between complement activation and elevated interleukin-6 and interleukin-8 levels in the NMP perfusate. Interestingly, complement activation was not observed in HMP, underscoring the temperature sensitivity of this response. Although the precise consequences of complement activation during NMP remain unclear, emerging studies suggest that targeting this pathway may have therapeutic potential. For example, complement inhibition in rodent models has shown promise in reducing graft injury, by downregulating key proteases such as C1s during NMP.^46^

#### Inflammation-Modulating Strategies

Efforts to dampen the proinflammatory milieu of NMP have included the administration of alpha-1-antitrypsin, a protease inhibitor with antiinflammatory effects. However, Mellati *et al.*^47^ reported no significant differences in cytokine expression or injury markers with alpha-1-antitrypsin treatment in porcine kidneys, whether delivered via postretrieval flush or during NMP.

Tissue-resident lymphocytes may also be influenced by NMP. Hullegie-Peelen *et al.*^48^ observed tissue-resident lymphocytes in the perfusate of NMP-preserved kidneys, likely because of the shedding of their adhesion partner, sE-cadherin, during ischemia-reperfusion injury. Although the functional implications of this tissue-resident lymphocytes displacement remain to be determined, it supports further studies on how donor immune memory within the graft may affect long term organ health.

### NMP as a Platform for Therapeutic Delivery

Beyond preservation, NMP offers a controlled platform to deliver targeted interventions directly to the organ, potentially enhancing graft function and modulating immunogenicity without systemic exposure. This approach introduces a new dimension of precision medicine where specific concerns on donor organ biopsy could be targeted prior to transplantation by pharmaceutical or cellular therapies. For example, complement inhibitors already approved for clinical use, such as C1 esterase inhibitors, have been shown in early trials to improve posttransplant outcomes when administered to high-risk kidneys before implantation.^49^, ^50^, ^51^ Although these studies were conducted with SCS, the real-time monitoring and extended exposure afforded by NMP could enable more nuanced and sequential targeting of the complement cascade.

Another therapeutic strategy under investigation is targeting fibrosis via the TGF-β pathway during NMP. In porcine models, this approach significantly reduced inflammation and fibrosis, with improved histological markers of graft viability.^52^ Likewise, mesenchymal stem cells have demonstrated regenerative, antiinflammatory, and antifibrotic effects in preclinical NMP studies, presenting an avenue for cell-based therapies.^53^, ^54^, ^55^

### Toward Immune Tolerance and Antigen Engineering

NMP provides a platform for next-generation therapies aimed at reshaping the immunogenicity of donor organs. Delivery of oligonucleotide-based agents to silence human leukocyte antigen expression has shown success in reducing alloimmune responses in rodent and porcine models.^56^^,^^57^ These strategies represent a potential step toward achieving immune tolerance and expanding transplantation options for sensitized recipients.

In addition, *ex situ* enzymatic modification of blood group antigens offers a novel strategy to overcome ABO incompatibility. Studies have demonstrated that bacteria-derived glycoside hydrolases can effectively convert donor kidneys to a universal blood group O phenotype, reducing antibody binding and complement activation.^58^^,^^59^ Although the durability of this approach remains to be validated, early antigen removal may promote immune accommodation even upon reexpression posttransplantation.^60^ Ongoing research into the underlying mechanisms and therapeutic opportunities of NMP will be essential to unlocking its full translational potential.

### NMP to Enhance Kidney Utilization Rates

Ultimately, the goal of kidney NMP is to improve preservation of donor organs and increase organ utilization. In 2024, > 8000 kidneys, or 25%, of all donor kidneys recovered in the US were ultimately discarded and not used for transplant.^27^ This issue stems from several factors, including inefficiencies in the organ allocation system, which often fails to match kidneys with transplant centers before the cold ischemia time surpasses acceptable limits, and an increased offer of marginal grafts which are even more susceptible to prolonged cold ischemia time.

The implementation of NMP has the potential to address these challenges by prolonging the allocation window and providing reassurance to transplant centers about the viability of a donor organ. One promising initiative is the ongoing OPTIMAL trial (A Central Preservation and Assessment Service to Optimize Donor Kidney Allocation, NCT06263023), a US multicenter study which seeks to evaluate whether transporting hard-to-place donor kidneys to a central facility for assessment with NMP can improve allocation success.^61^ In this trial, hard-to-place donor kidneys are transported to a central facility and placed on NMP for a brief evaluation period. Parameters such as intrarenal resistance, oxygen consumption, and electrolyte balance are measured, and the QAS^39^ is used to assess kidney viability (Table 2). Kidneys deemed suitable for transplantation are subsequently transported to recipient centers using a portable oxygenated HMP device.

The trial enrollment has been completed, and while the final results are pending publication, 80 previously hard-to-place kidneys were successfully rescued and transplanted across multiple US centers within a 1-year period.^61^ This achievement underscores the potential of this approach to alleviate organ allocation challenges, particularly by enabling the transfer of kidneys from regions with greater availability to those with longer waitlists.

### Normothermic Regional Perfusion: Complementary to NMP or an Alternative Approach?

Normothermic regional perfusion (NRP) is an increasingly adopted technique in DCD organ donor recovery protocols.^62^ By restoring *in situ* circulation to the organs via cardiopulmonary bypass or extracorporeal membrane oxygenation after death has been declared, NRP allows for additional assessment and optimization prior to organ retrieval. This approach may mitigate the effects of warm ischemia and may increase the pool of transplantable organs while improving outcomes. Abdominal NRP is used to better preserve the liver and kidneys for transplant.^63^

A recent systematic review by Klein Nulend *et al.*^64^ reported lower rates of DGF in DCD kidneys subjected to NRP compared to those undergoing NMP, though overall graft outcomes were comparable. Each technique presents distinct advantages and limitations. NRP can be initiated directly at the donor hospital and simultaneously support multiple organs; however, its duration is typically limited and organ-specific evaluation, particularly for kidneys, remains challenging. In addition, the organs remain within the pro-inflammatory milieu of a deceased donor. Conversely, NMP allows for extended perfusion and individualized kidney evaluation and treatment, albeit with greater logistical demands.

Ultimately, the selection of an appropriate perfusion strategy—whether HMP, NRP, or NMP—should be guided by donor characteristics, logistical considerations, and intended therapeutic goals. As perfusion technologies evolve, selecting the appropriate modality will be critical to maximizing graft utilization and improving transplant outcomes for deceased donor kidneys.

### Conclusion

Kidney NMP represents a promising advancement in organ preservation, with growing evidence supporting its potential to improve graft assessment, allocation, and posttransplant outcomes. However, further research is needed to optimize and standardize perfusates and perfusion parameters before widespread clinical adoption. Beyond preservation, NMP offers a unique platform for modulating graft immunogenicity and promoting regeneration before transplantation. As the field advances, ethical considerations, particularly around discard criteria, consent, access, and the use of marginal organs, must be carefully addressed. Clear communication and robust ethical oversight will be crucial to ensure equitable and responsible use. Ultimately, refining this technology could reduce organ discard rates, enhance long-term graft survival, and expand transplant access for highly sensitized patients (Figure 2).

**Figure 2:**
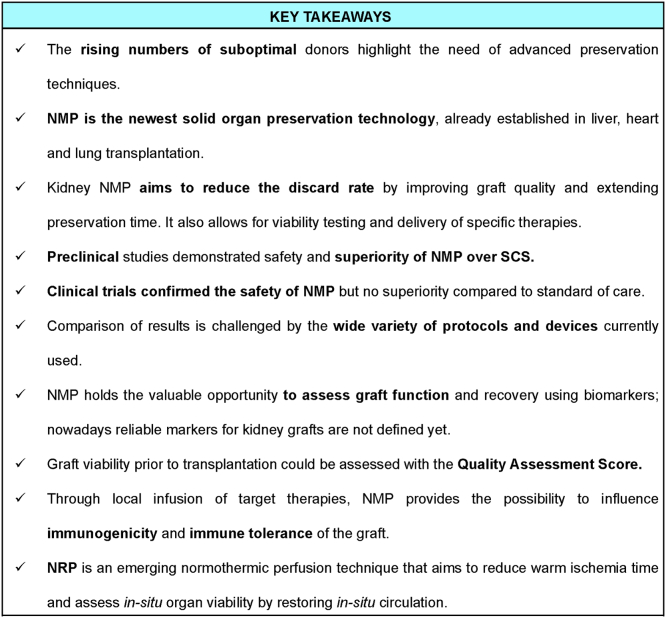
Key takeaways. NMP, normothermic kidney perfusion; NRP, normothermic regional perfusion; SCS, static cold storage.

## Disclosure

All the authors declared no competing interests.
